# Full‐Space Terahertz Airy Beam Shaping via Composite Bilayer Metasurface With Switchable Amplitude–Phase Encoding Channels

**DOI:** 10.1002/nap2.70351

**Published:** 2026-10-31

**Authors:** Yunting Bai, Shaohe Li, Yan Wang, Qike Shi, Fan Nie, Xin Jue, Yujie Liu, Hailin Wang, Runfeng Su, Jian Chen

**Affiliations:** ^1^ Yanzhao Electric Power Laboratory of North China Electric Power University North China Electric Power University Baoding Hebei China; ^2^ Hebei Engineering Research Center of Intelligent Technology for Power Internet of Things North China Electric Power University Baoding Hebei China; ^3^ Research Institute of Superconductor Electronics School of Electronic Science and Engineering & National Key Laboratory of Microwave Photonics (Nanjing University) Nanjing University Nanjing Jiangsu China; ^4^ National Key Laboratory of Microwave Photonics College of Electronic and Information Engineering Nanjing University of Aeronautics and Astronautics Nanjing China; ^5^ State Key Laboratory of Millimeter Waves Southeast University Nanjing China

**Keywords:** airy beams, frequency multiplexing, near‐field wavefront engineering, polarization multiplexing, terahertz metasurfaces

## Abstract

Terahertz (THz) Airy beams hold significant promise for directional wireless links in future 6G communications. Unlike conventional phased arrays with high power consumption and complexity, metasurfaces offer a platform for flexibly tailoring the Airy beam. However, existing THz metasurface‐based Airy beam generators remain confined to one dimension, and experimental verification of their quasi‐nondiffracting and self‐healing characteristics is scarce. Here, we propose a composite bilayer metasurface for full‐space THz Airy beam manipulation and experimentally demonstrate its transmission channel capabilities. By varying the structural rotation angles, the metasurface enables linearly polarized (LP) amplitude‐phase and circularly polarized (CP) Pancharatnam‐Berry (PB) phase, switching between distinct transmission channels at 0.99 THz and reflection channels at 0.538 and 0.553 THz. Ring Airy beams (RAB), phase‐modulated ring Airy beams (PRAB), and elliptical Airy vortex beams (EAVB) are realized based on the proposed composite bilayer architecture. As proof‐of‐concept demonstrations, the prototypes of LP RAB and CP EAVB are fabricated and experimentally verified. The measured distributions show good agreement with simulations, confirming the ability of the metasurfaces to manipulate Airy beams across polarization states, frequencies, and transmission directions. This work provides a compact route toward multi‐dimensional THz Airy beam manipulation with broad application prospects in areas such as free‐space optical communications and 6G THz wireless communication.

## Graphical abstract

A composite bilayer metasurface integrates linear‐polarization amplitude–phase modulation with circular‐polarization Pancharatnam–Berry phase modulation, enabling multidimensional terahertz Airy beam manipulation within a single compact aperture. The highly integrated platform supports diverse Airy beam generation and offers new opportunities for multifunctional wavefront engineering in free‐space optics and future 6G wireless communications.

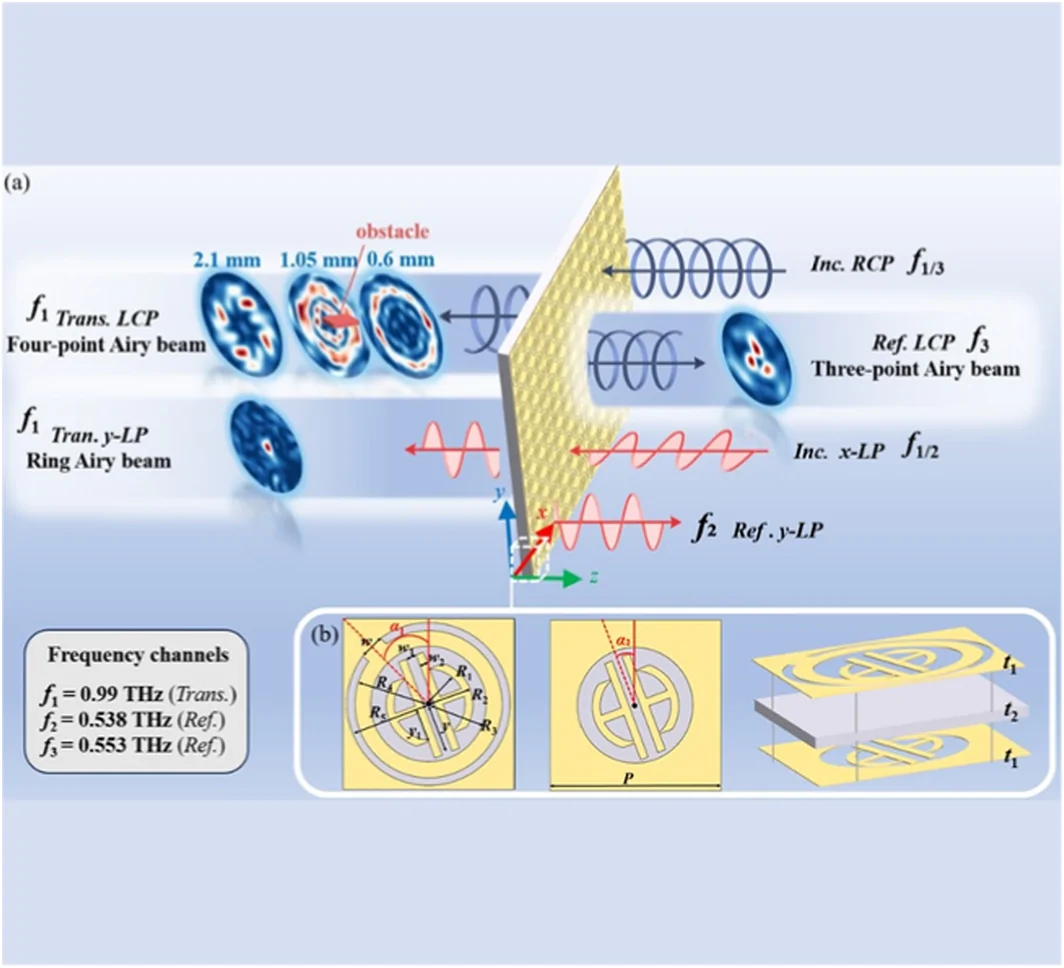

## 1 Introduction

The terahertz (THz) band, with abundant spectral resources and large potential transmission capacity, is regarded as an important candidate frequency range for future 6G wireless communications [1, 2, 3]. However, THz waves still suffer from large free‐space path loss, limited source power, and high sensitivity to blockage, making stable transmission and flexible energy delivery challenging in complex environments [4, 5, 6, 7, 8, 9, 10, 11]. Directional wireless links based on conventional collimated or focused beams can improve directivity and link gain, but their spatial field distributions and propagation paths remain relatively fixed. Hence, these beams are not suitable for complex beam‐control applications in scenarios such as local obstruction, multi‐receiver energy delivery, and near‐field wavefront control [7, 8, 12]. Therefore, developing beam‐control devices capable of tailoring the spatial propagation behavior of electromagnetic (EM) fields is important for improving the adaptability of THz wireless links in complex propagation environments [12, 13, 14, 15, 16, 17, 18, 19, 20].

Airy beams are characterized by quasi‐nondiffracting propagation, self‐bending trajectories, and self‐healing behavior [21, 22, 23, 24, 25, 26], which allow them to propagate along curved paths and reconstruct the main field distribution after partial obstruction [24, 25, 26]. Such properties render them attractive for obstacle‐avoiding transmission, near‐field energy reconstruction, and complex‐path beam control. Conventional approaches for Airy beam generation usually rely on spatial light modulators, phase plates, computer‐generated holograms, or phased‐array‐based systems [27, 28]. Although these methods can generate prescribed beam profiles, they still face limitations in several aspects, including device compactness, feeding‐network complexity, power consumption, and system integration, especially in the THz band.

Metasurfaces composed of subwavelength artificial elements can manipulate the phase, amplitude, and polarization of EM waves within a planar structure, providing an effective route for tailoring the spatial evolution of THz Airy beams [29, 30, 31, 32, 33]. At THz frequencies, amplitude‐phase modulation has been realized by combining propagation and geometric phases [34], employing spin‐decoupled dual‐channel complex‐amplitude encoding [35], and independently controlling the geometry and orientation of multilayer meta‐atoms [36]. Moreover, related studies at microwave frequencies have been demonstrated through polarization decomposition and routing [37], frequency‐direction multiplexing [38], hybrid‐phase‐assisted full‐space modulation [39], and chirality‐assisted independent control of phase, amplitude, and polarization [40]. These developments provide a foundation for composite metasurface designs that integrate multiple electromagnetic modulation functionalities within a compact aperture.

Despite these advances, experimental demonstrations of THz metasurface‐based Airy beams have mainly focused on single‐beam generation or the verification of fundamental propagation characteristics [41, 42, 43, 44]. The independent assignment of diverse Airy beam functionalities to distinct polarization, frequency, and transmission/reflection channels within a single compact metasurface aperture remains insufficiently explored. This capability is relevant to compact indoor and data‐center near‐field THz wireless links, where multiple receivers may be located at different positions on the common receiving plane. The multi‐spot beam control is relevant to parallel spatial addressing and spatially distributed coverage in multi‐user near‐field THz links [45, 46, 47], with potential relevance to broadcast and spatial‐diversity applications. Compared with multiple separately designed beam‐forming elements or independently aligned components, a shared‐aperture metasurface for multi‐spot Airy‐beam generation integrates several beam functionalities into a compact device and can reduce alignment requirements for links with different receiver positions or propagation directions. These considerations motivate the composite metasurface developed in this work.

In this work, we propose a composite bilayer metasurface for multidimensional THz Airy beam manipulation. By tailoring the rotation angle of the structural elements, linear polarization (LP) amplitude‐phase modulation and circular polarization (CP) Pancharatnam–Berry (PB) phase modulation are implemented in the transmission and reflection channels at different operating frequencies. Based on this strategy, the metasurface supports diverse Airy beams, including ring Airy beams (RAB), phase‐modulated ring Airy beams (PRAB), and elliptical Airy vortex beams (EAVB). For experimental demonstration, three representative metasurface arrays are fabricated for Airy beam generation in the transmission channel: two for generating LP RABs and one for generating a CP EAVB. The measured near‐field patterns reproduce the characteristic beam features predicted by the corresponding transmission simulations. In contrast, the generation of Airy beams in the reflection channel is demonstrated through numerical simulations. These results offer a practical pathway toward compact THz near‐field devices with controllable structured‐beam evolution.

## 2 Design of the Composite Bilayer Meta‐Atom

The conceptual illustration of the proposed composite bilayer metasurface is shown in Figure 1a. Under the right‐handed circular polarization (RCP) wave incidence, the metasurface produces a left‐handed circular polarization (LCP) four‐point PRAB in the transmission channel at *f*_1_ = 0.99 THz and a three‐point PRAB in the reflection channel at *f*_3_ = 0.553 THz. The electric field evolution shown on the transmission channel further illustrates the self‐healing behavior of the four‐point PRAB after partial obstruction. For *x*‐LP wave incidence, the *y*‐LP RAB is generated in the transmission channel at *f*_1_ = 0.99 THz, while a *y*‐LP reflected wave is obtained at *f*_2_ = 0.538 THz.

**FIGURE 1:**
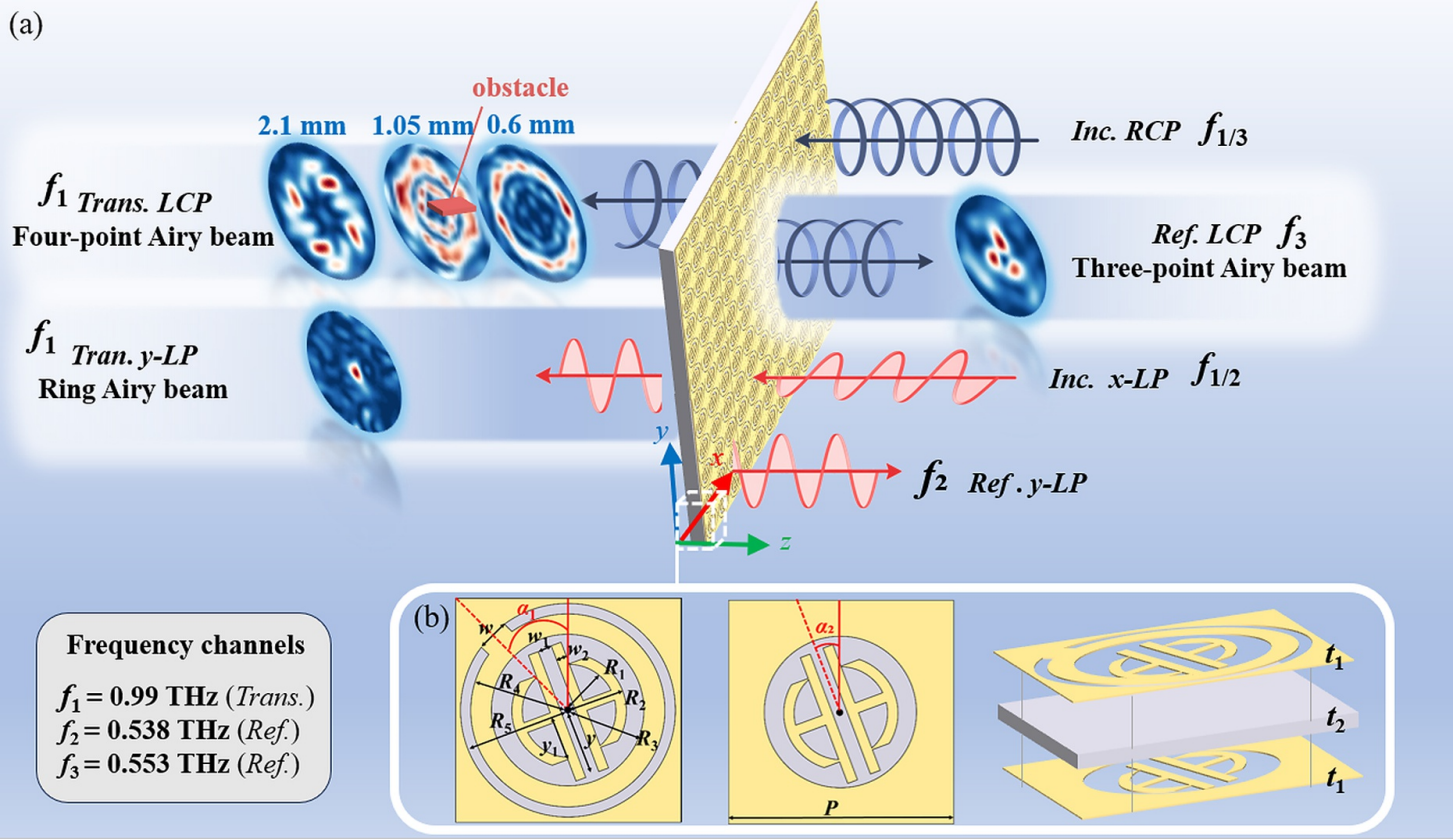
(a) Conceptual illustration of the proposed composite bilayer metasurface. (b) Schematic of the proposed meta‐atom: top view, bottom view, and side view. The geometric parameters of the meta‐atom (all dimensions are in μm) are *p =* 130, *w* = 5.5, *w*_1_ = 10, *w*_2_ _=_ 6, R_1_ = 27, *R*_2_ _=_ 33, *R*_3_ _=_ 44, *R*_4_ _=_ 56, *R*_5_ = 62, *y =* 26, *y*_1_ _=_ 39, *t*_1_ = 0.2, *t*_2 =_ 50.

These multi‐dimensional modulation capabilities are enabled by the designed composite meta‐atom, whose top, bottom, and side views are presented in Figure 1b with detailed geometric parameters. The meta‐atom consists of two gold metallic layers separated by a polyimide dielectric layer. In the simulations, gold is modeled as a lossy metal with an electrical conductivity of *σ* = 4.561 × 10^7^ S/m, and the polyimide substrate is characterized by a dielectric constant of *ε* = 3.5 and a loss tangent of tan *δ* = 0.0027. In this configuration, the central *Φ*‐shaped metallic structures patterned on both the top and bottom layers mainly govern the transmission channel response, whereas the outer C‐shaped aperture patterned on the top layer primarily controls the reflection channel response. In addition, a via‐hole‐assisted frequency‐selective structure is introduced to tailor the electromagnetic (EM) responses at different frequencies, enabling the separated modulation of the transmission/reflection channels within a single meta‐atom.

Beyond this channel‐selective structural design, the proposed meta‐atom also supports two distinct polarization modulation mechanisms. Under the LP wave incidence, the modulation is dominated by the cross‐polarized component, which exhibits a sin (2*α*) type dependence on the rotation angle of the anisotropic meta‐atom. Under the CP incidence, the anisotropic meta‐atom provides PB phase modulation. The phase of the cross‐polarized output follows the relation *Φ* = ± 2*α*, enabling continuous phase control through in‐plane rotation. A detailed Jones matrix analysis of the LP and CP modulation mechanisms is provided in Supporting Information S1: Section S1 of the Supplementary Materials.

The electromagnetic responses of the meta‐atoms are simulated using the time‐domain solver in CST Studio Suite. Periodic boundary conditions are applied along the *x*‐ and *y*‐directions to represent an infinitely periodic array, while open boundaries are imposed along the *z*‐direction. Floquet ports are used along the *z*‐direction to excite and collect the fundamental mode. The polarization is defined with respect to the wave‐propagation direction following the convention implemented in CST Studio Suite.

Figure 2a shows the cross‐polarized transmission response *t*_*yx*_ and *Φ*_*tyx*_ under *x*‐polarized wave incidence within the frequency range of 0.7–1.3 THz. The three‐dimensional amplitude map exhibits a ridge, and the maximum cross‐polarized amplitude reaches approximately 0.8. At the operating frequency *f*_1_ = 0.99 THz, the normalized amplitude profile shows a monotonic increase from zero at *α*_2_ = 0° to a maximum at *α*_2_ = ± 45°, following an approximate sin (2*α*_2_) dependence. Meanwhile, the phase shows a binary response, with a 180° phase shift between positive and negative rotation angles. At 0.99 THz, the polarization‐conversion ratio (PCR) is 91.99%, and the total transmission is 78.43%, varying from 78.10% to 83.19% over the defined operating bandwidth of 0.947–1.027 THz.

**FIGURE 2:**
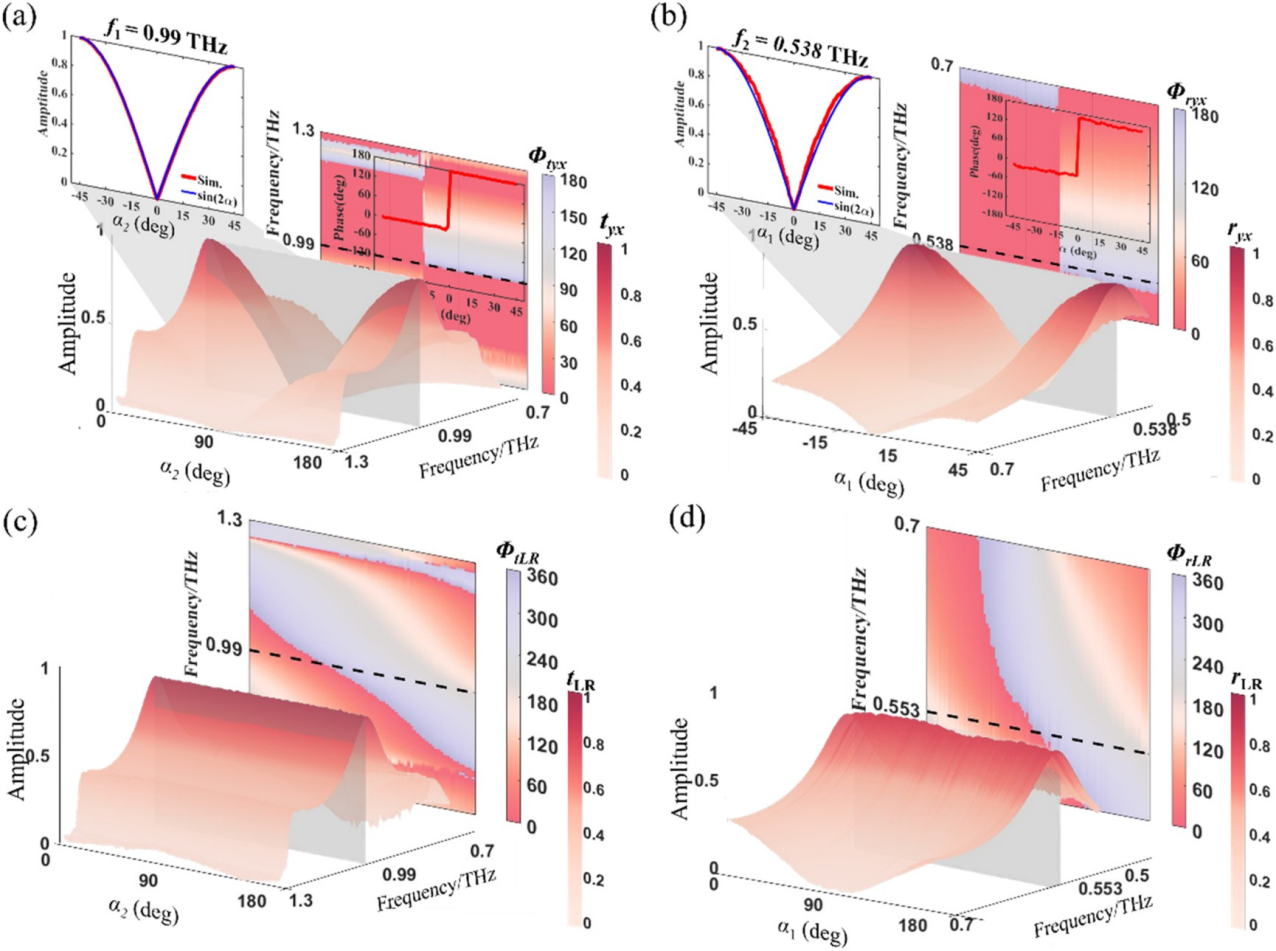
The amplitude and phase modulation characteristics of the proposed composite meta‐atom. (a) Cross‐polarized transmission coefficient *t*_yx_ and phase *Φ*_*tyx*_ under *x*‐polarized wave incidence, with the inset showing the amplitude at 0.99 THz. (b) Cross‐polarized reflection coefficient *r*_yx_ and phase *Φ*_ryx_ under *x*‐polarized wave incidence, with the inset showing the amplitude at 0.538 THz. (c) Cross‐polarized transmission coefficient *t*_*LR*_ and phase *Φ*_*tLR*_ under RCP wave incidence. (d) Cross‐polarized reflection coefficient *r*_*LR*_ and phase *Φ*_*rLR*_ under RCP wave incidence.

A similar trend is observed in the reflection channel. The cross‐polarized reflection coefficient *r*_*yx*_ and *Φ*_*ryx*_ over the frequency range of 0.5–0.7 THz are plotted in Figure 2b. The cross‐polarized reflection coefficient reaches a maximum of 0.8 at *f*_2_ = 0.538 THz. The normalized response follows an approximate sin (2*α*_1_) dependence, while the phase exhibits a binary behavior with a 180° difference between positive‐ and negative‐angle branches. At 0.538 THz, the PCR is 92.79%, and the total reflection is 65.87%, with a maximum value of 66.97%. The corresponding operating bandwidth is 0.529–0.549 THz.

For RCP wave incidence, Figure 2c presents the transmission coefficient *t*_*LR*_ and *Φ*_*tLR*_ from 0.7 to 1.3 THz. A pronounced amplitude peak appears at *f*_1_ = 0.99 THz. Meanwhile, the phase distribution exhibits a nearly linear dependence on the rotation angle over the 0°–180° range, which is characteristic of PB phase modulation. At 0.99 THz, the PCR is 88.73%, and the total transmission is 80.08%, varying from 79.41% to 80.58% over the operating bandwidth of 0.951–1.028 THz.

The same PB phase mechanism is also observed in the reflection channel. As presented in Figure 2d, the reflection coefficient *r*_LR_ maintains a relatively high amplitude at *f*_3_ = 0.553 THz over the frequency range of 0.5–0.7 THz. Meanwhile, the phase *Φ*_rLR_ varies linearly with the rotation angle, following *Φ* = 2*α*_1_. At 0.553 THz, the PCR is 81.18%, and the total reflection is 68.83%, varying from 66.86% to 70.90% over the operating bandwidth of 0.530–0.554 THz. The calculation procedures for the PCR and total power are provided in Supporting Information S1: Section S1 of the Supplementary Materials.

To further clarify the physical origin of the multiplexed response, the isolation between the transmission and reflection channels is analyzed through surface current distributions and parameter‐dependent responses in Supporting Information S1: Section S2 of the Supplementary Materials. The resulting low crosstalk indicates limited mutual interference between the channels and provides a basis for multifunctional wavefront engineering.

## 3 Results and Discussion

### 3.1 Phase‐Modulated Ring Airy Beam

A conventional RAB wavefront typically produces one autofocusing point. This configuration is suitable for a point‐to‐point near‐field link because the available aperture power is concentrated at one receiver position. However, it does not directly provide multiple independently controlled focal regions for simultaneously addressing receivers located on the same receiving plane. To address this limitation, an azimuthal phase modulation strategy is introduced to split the RAB into multiple focal points with controllable point numbers and balanced energy distributions. The required electric field distribution for PRAB modulation at the initial plane *z* = 0 is given by:

(1) $$E_{\text{PRAB}}\left(r,\theta \right)=C\times Ai\left(r_{0}-r/b\right)\times \exp\left(a\left(r_{0}-r/b\right)\right)\times f\left(\theta \right),$$

where *Ai* (·) denotes the Airy function. *C* is a constant amplitude coefficient, *r*_*0*_ determines the radius of the main ring, *b* controls the radial scale of the Airy profile, and *a* is the exponential truncation parameter introduced to obtain a finite‐energy beam. The angular modulation function *f*(*θ*) is defined as:

(2) $$f\left(\theta \right)=\exp\left(im\theta \right)\times \gamma_{N}\left(\theta \right).$$

The exp (*imθ*) is the helical phase term, *m* denotes the vortex topological charge, and *θ* is the azimuthal coordinate. The term $\gamma_{N}\left(\theta \right)$ represents the azimuthally spliced power‐exponential phase distribution, which introduces discrete angular phase modulation into the original rotationally symmetric RAB wavefront. By controlling the angular phase distribution, γ(*θ*) determines the number and azimuthal arrangement of the generated focal spots. The specific forms for the three‐point and four‐point configurations are expressed as:

(3) $$\gamma_{3}\left(\theta \right)=\left\{\begin{array}{l} \exp\left\{i\delta {\left[\left(\theta -\pi /3\right)/2\pi \right]}^{2}\right\},\;0\leq \theta <2\pi /3\\ \exp\left\{i\delta {\left[\left(\theta -\pi \right)/2\pi \right]}^{2}\right\},\;2\pi /3\leq \theta <4\pi /3\\ \exp\left\{i\delta {\left[\left(\theta -5\pi /3\right)/2\pi \right]}^{2}\right\},\;4\pi /3\leq \theta <2\pi \end{array}\right.,$$

(4) $$\gamma_{4}\left(\theta \right)=\left\{\begin{array}{l} \exp\left\{i\delta {\left[\left(\theta -\pi /4\right)/2\pi \right]}^{2}\right\},\;0\leq \theta <\pi /2\\ \exp\left\{i\delta {\left[\left(\theta -3\pi /4\right)/2\pi \right]}^{2}\right\},\;\pi /2\leq \theta <\pi \\ \exp\left\{i\delta {\left[\left(\theta -5\pi /4\right)/2\pi \right]}^{2}\right\},\;\pi \leq \theta <3\pi /2\\ \exp\left\{i\delta {\left[\left(\theta -7\pi /4\right)/2\pi \right]}^{2}\right\},\;3\pi /2\leq \theta <2\pi \end{array}\right..$$

In an unmodulated RAB, the electric field exhibits continuous rotational symmetry, and the angular components interfere to produce a rotationally symmetric beam. The azimuthal phase modulation breaks this symmetry by imposing different phase delays at different azimuthal positions. During propagation, these components interfere constructively at selected positions and destructively elsewhere, transforming the continuous focal ring into several discrete focal points. The parameter *δ* controls the phase‐gradient strength of the azimuthal modulation, thereby affecting the angular phase accumulation, focal‐point separation, and relative energy distribution. The integer *m* denotes the vortex topological charge and determines the phase winding around the beam axis, thereby controlling the size and structure of the central dark core. Together, *δ* and *m* determine the spatial arrangement, energy uniformity, and sidelobe level of the generated PRAB.

As proof‐of‐concept demonstrations, a three‐point PRAB is generated in the reflection channel using *γ*_3_(*θ*), *δ* = −20π, and *m*_3_ = 1, whereas a four‐point PRAB is generated in the transmission channel using *γ*_4_(*θ*), *δ* = −10π, and *m*_4_ = 5. Here, the subscripts 3 and 4 in *m*_3_ and *m*_4_ denote the three‐point and four‐point PRAB configurations, respectively, rather than different definitions of the topological charge. Based on the systematic parameter analysis in Supporting Information S1: Section S3 of the Supplementary Materials, the selected combinations provide clearly resolved focal points, relatively uniform energy distributions, and weak sidelobes under the finite‐aperture condition. The remaining parameters are kept identical, with *r*_0_ = 7.5 × 10^−4^, *b* = 1.5 × 10^−4^, and *a* = 0.001.

For PRAB generation, the three‐point and four‐point configurations are assigned to the reflection and transmission channels, respectively. The corresponding target fields are defined by Equations ((1), (2), (3), (4)), and their phase distributions are sampled to obtain the phase profiles of the discretized metasurface arrays, as shown in Figure 3a,b. To characterize the ideal beam evolution, the normalized longitudinal electric‐field intensity distribution in Figure 3c is calculated from the continuous target fields using scalar diffraction theory. By contrast, the transverse electric‐field distributions in Figure 3d–i are obtained from full‐wave EM simulations of the corresponding finite metasurface arrays. These simulations are performed using a time‐domain solver with open (add space) boundary conditions applied in all directions. The metasurfaces are illuminated by a plane wave propagating along the ‐*z* direction. During propagation, the PRABs gradually split into three or four lobes. These lobes move inward and converge at their corresponding focal plane, followed by beam divergence beyond the focus.

**FIGURE 3:**
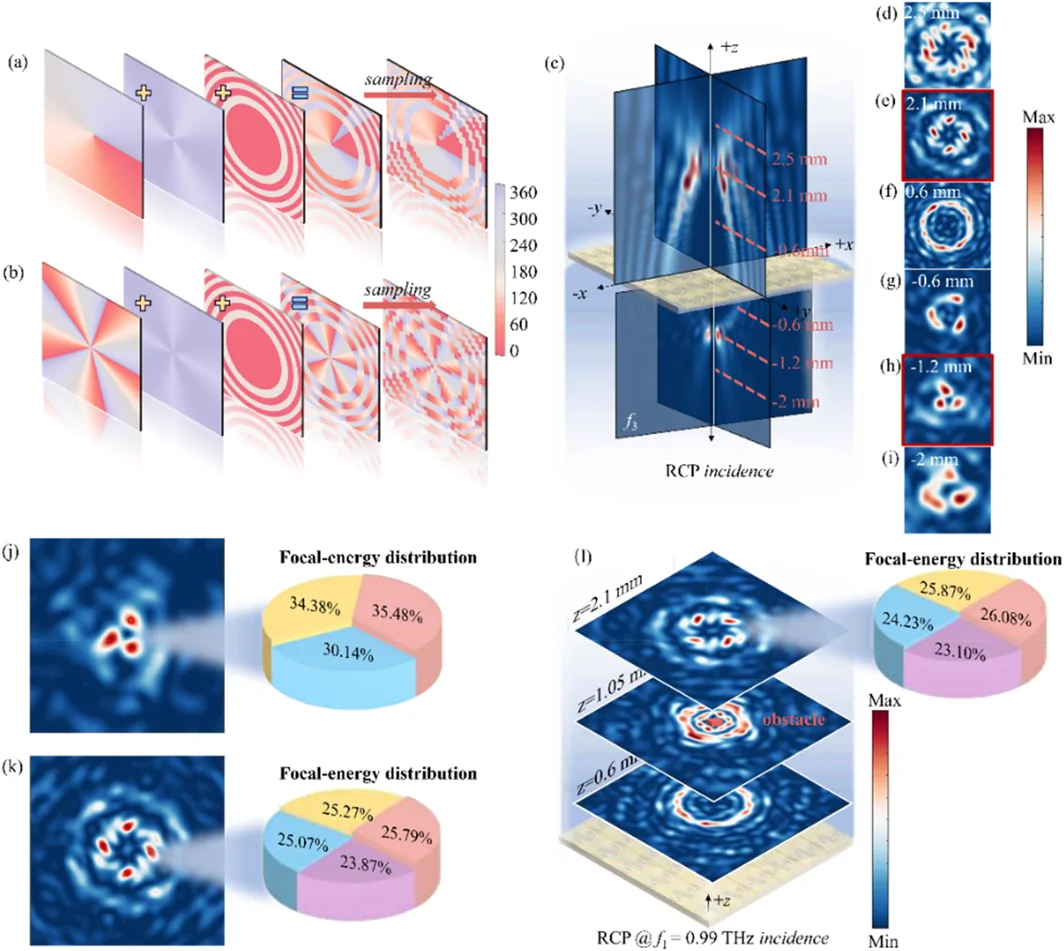
Characterization of the PRAB metasurfaces. The three‐point PRAB operates in the reflection channel at 0.553 THz, whereas the four‐point PRAB operates in the transmission channel at 0.99 THz. Calculated phase profiles of the (a) three‐point and (b) four‐point PRAB. (c) Numerically simulated normalized electric field intensity distributions in the longitudinal planes. Full‐wave EM simulated normalized electric field intensity distributions at propagation distances of (d) 2.5 mm, (e) 2.1 mm, (f) 0.6 mm, (g) −0.6 mm, (h) −1.2 mm, and (i) −2.0 mm, respectively. (j) Energy proportions of the three‐point PRAB and (k) the four‐point PRAB. (l) Full‐wave EM simulated self‐healing behavior of the four‐point PRAB.

To evaluate the focal electric field quality, the energy proportion of each focal point is calculated by integrating |*E* (*x*,*y*)|^2^ within a circular region with a radius of 60 μm centered at its intensity maximum and normalizing the result by the summed integrated energies of all focal points. As shown in Figure 3j,k, the three‐point PRAB exhibits energy proportions of 35.70%, 34.02%, and 30.28%, while the four‐point PRAB yields 25.62%, 25.35%, 25.07%, and 23.95%. The small residual imbalance mainly arises from the finite 30 × 30 square aperture, discrete sampling of the continuous amplitude and phase profiles, and inter‐element coupling. Despite these nonidealities, the calculated proportions remain close to the ideal values of 33.33% and 25.00% for the three‐ and four‐point PRAB, respectively. The target polarization relative focusing efficiencies reach 35.05% and 44.60% for the four‐point transmission PRAB and three‐point reflection PRAB, respectively. These results confirm efficient concentration of the desired polarization component at the prescribed focal spots. The calculation method for the focusing efficiencies is provided in Supporting Information S1: Section S3 of the Supplementary Materials.

The self‐healing property is further examined using the four‐point PRAB as a representative case. A metallic obstacle (100 μm × 100 μm × 2 μm) is placed at *z* = 1 mm, and the resulting field evolution is shown in Figure 3l. Although the four‐point PRAB is initially disrupted, it gradually reconstructs during propagation and is restored near the focal plane at *z* = 2.1 mm. The reconstructed focal points exhibit energy proportions of 26.08%, 25.87%, 23.10%, and 24.24%, remaining close to the unobstructed case. These results confirm that the PRAB preserves the self‐healing characteristic while maintaining a robust and balanced multifocal distribution.

### 3.2 Elliptical Airy Vortex Beam

Unlike the radially symmetric RAB, the EAVB introduces an ellipticity‐induced symmetry breaking into the Airy‐type field, leading to an intrinsically asymmetric focal topology [46]. Moreover, the asymmetric intensity distribution has been reported to improve resilience against atmospheric turbulence. The required electric field of the EAVB metasurface is given by Equation (5).

(5) $$\begin{array}{l} E_{\text{EAVB}}\left(x_{0},y_{0},0\right)=C\times Ai\left(\left(r_{0}-\sqrt{x_{0}^{2}+t^{2}y_{0}^{2}}\right)/b\right)\\ \times \;\exp\left(a\left(r_{0}-\sqrt{x_{0}^{2}+t^{2}y_{0}^{2}}\right)/b\right)\times \exp\left(im\theta \right) \end{array},$$

where *r*_0_ and *b* retain the definitions given in Section 3.1 and are set to *r*_0_ = 7.5 × 10^−4^ and *b* = 1.5 × 10^−4^, respectively. The parameter *t* = 0.7 determines the ellipticity of the beam, *m* denotes the vortex topological charge, and *θ* = arctan (*y*/*x*) is the azimuthal coordinate.

For EAVB generation, two transmission‐mode metasurfaces are designed with topological charges *m* = 2 and *m* = 3. The corresponding phase profiles are obtained by sampling the phases of the theoretical target fields defined by Equation (5) and discretizing them on 55 × 55 meta‐atom arrays, as shown in Figure 4a,b. The metasurface aperture covers the spatial range *x*_*0*_, *y*_*0*_ ∈ [−3.575, 3.575] mm. To characterize the ideal beam propagation, the normalized longitudinal electric‐field intensity distributions in the *xz*‐ and *yz*‐planes, shown in Figure 4c,f, are calculated by propagating the corresponding target fields using the scalar diffraction method.

**FIGURE 4:**
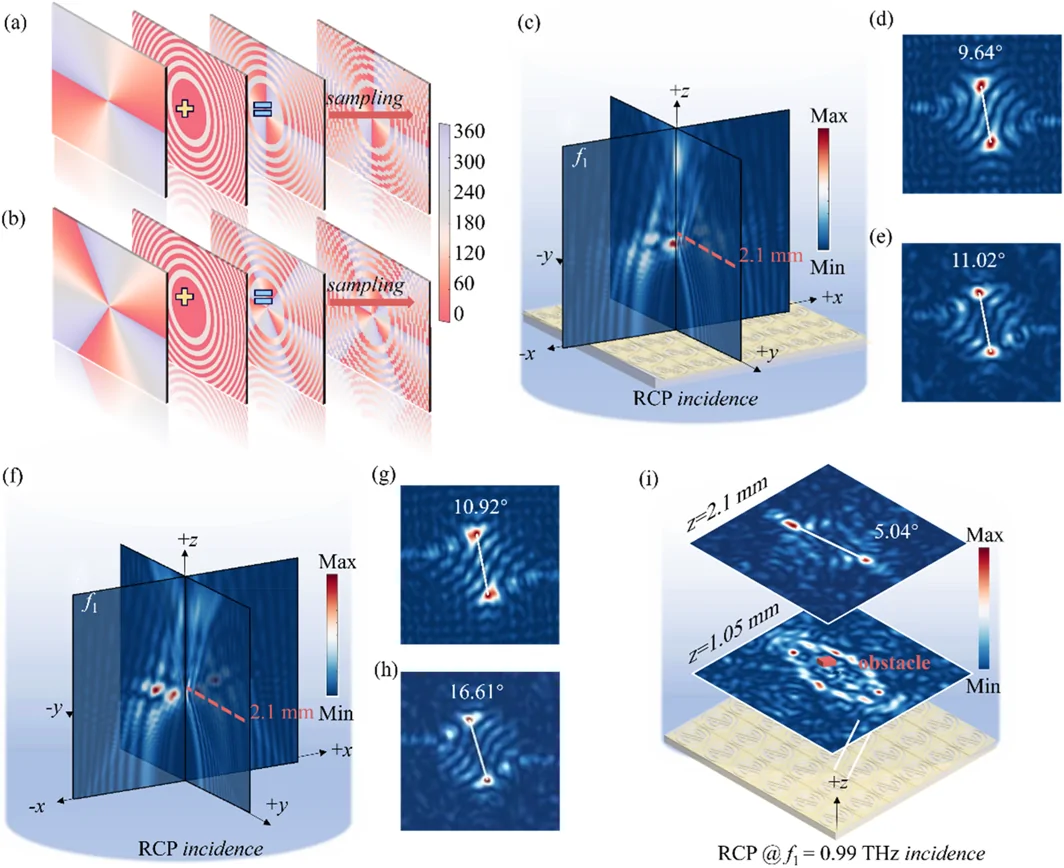
Characterization of the EAVB metasurfaces in the transmission channel at 0.99 THz. Calculated phase profile of the EAVBs with the topological charge (a) *m* = 2 and (b) *m* = 3. Numerically simulated electric field propagation characteristics of the EAVBs with the topological charge (c) *m* = 2 and (f) *m* = 3 in the *xz*‐ and *yz*‐planes. (d) Numerically simulated and (e) Full‐wave EM simulated normalized electric intensity distributions of the EAVB with the topological charge *m* = 2 in the *xy*‐plane. (g) Numerically simulated and (h) Full‐wave EM simulated normalized electric intensity distributions of the EAVB with the topological charge *m* = 3 in the *xy*‐plane. (i) Full‐wave EM simulated self‐healing behavior of the EAVB with the topological charge *m* = 2.

The corresponding transverse electric‐field intensity distributions at the focal plane *z* = 2.1 mm are shown in Figure 4d,g. For comparison, Figure 4e,h present the full‐wave EM simulation results for the corresponding discretized phase‐only metasurface arrays. These EM simulations employ the same solver, boundary conditions, and plane‐wave excitation as those described in Section 3.1. Consistent with the theoretical prediction of |*m*|–1 secondary beams, the case of *m* = 2 exhibits two primary beams and one secondary beam, while the *m* = 3 case generates two secondary beams. The dependence of the beam tilt direction on the sign of *m* is further discussed in Supporting Information S1: Section S4 of the Supplementary Materials, which shows that the sign of *m* determines the direction of the beam orientation.

To quantify the asymmetric focusing behavior, the tilt angle is extracted from the line connecting the intensity maxima of the two dominant lobes. For the *m* = 2 EAVB, the extracted tilt angle is 9.64° in the numerical simulation and 11.02° in the EM simulation. For the *m* = 3 EAVB, the corresponding values increase to 10.92° and 16.61°. This difference arises from the increased deviation between the ideal continuous target field and the physically implemented finite phase‐only metasurface, with additional contributions from finite‐aperture truncation, discrete phase sampling, and nonideal meta‐atom responses. The smaller deviation observed for the *m* = 2 EAVB can be attributed to its relatively simpler phase distribution, which is less sensitive to phase discretization and finite‐aperture effects.

The self‐healing property is further verified by introducing an identical obstacle. As shown in Figure 4i, the beams are initially obstructed and then reconstructed during propagation, confirming the self‐healing behavior of the EAVB. Together with previous studies of Airy beam transmission, acceleration, self‐healing, and obstacle‐tolerant THz links [2, 22, 23, 25, 26], the numerical results presented here highlight the potential of Airy beams for wireless communication in obstructed propagation environments.

### 3.3 Fabrication and Experimental Characterization

Four metasurface samples are fabricated and experimentally characterized: one periodic meta‐atom array for S‐parameter characterization, two RAB‐generating arrays detailed in Supporting Information S1: Section S8 of the Supplementary Materials, and one EAVB‐generating array described in Section 3.2 of the main text. The resonators are redesigned with a uniform arm width to improve fabrication tolerance. The detailed fabrication process involving spin coating, thermal curing, photolithography, magnetron sputtering, and lift‐off is provided in Supporting Information S1: Section S5 of the Supplementary Materials.

Since the available THz time‐domain spectroscopy (THz‐TDS) system is configured for transmission‐mode near‐field scanning, the experimental characterization in this work is limited to the transmission channel. Reflection‐mode characterization requires reconfiguring the optical path and adopting a dedicated same‐half‐space measurement configuration capable of separating the incident and reflected waves. Since the transmission and reflection channels share the same physical mechanism, the present numerical reflection results together with the experimental transmission results are sufficient to confirm that reflection‐mode operation is achievable. Accordingly, the reflection Airy beam functionalities are evaluated numerically in the present work, whereas experimental validation in reflection mode will be pursued in future work.

Under the LP wave incidence, the transmission coefficients are measured using a THz‐TDS system as shown in Supporting Information S1: Section S6 of the Supplementary Materials. Two orthogonal sample orientations are used to emulate the rotation states of *α*_2_ = −45° and 45°, as shown in Supporting Information S1: Section S7 of the Supplementary Materials. As shown in Figure 5a, the measured amplitude agrees well with the trend in Figure 2, and the phase shows a clear 180° phase difference. The spectral response near 0.8 THz verifies the amplitude‐ and phase‐modulation characteristics of the fabricated meta‐atom but does not determine the beam‐generation frequency. Specifically, the periodic sample characterizes a uniform meta‐atom array, whereas beam generation depends on the collective amplitude and phase response of the complete finite metasurface. Therefore, the characteristic frequency of the periodic array does not necessarily coincide with the frequency at which the beam profile is observed.

**FIGURE 5:**
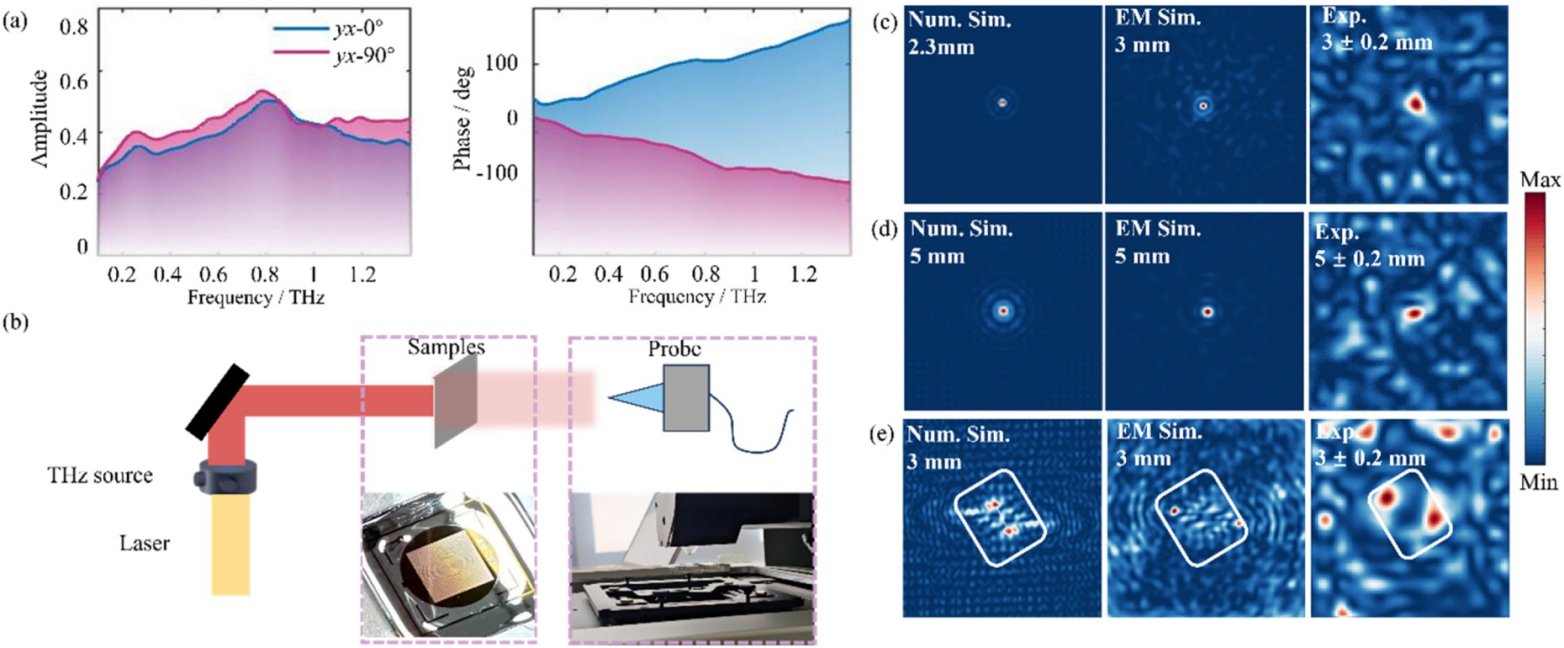
Experimental characterization of the fabricated metasurface samples in the transmission channel. (a) Measured cross‐polarized transmission coefficients under *x*‐polarized wave excitation. (b) Schematic of the THz near‐field scanning setup. The numerically simulated, full‐wave EM simulated, and experimental normalized electric field distributions *E*_*y*_ in the transmission channel of the RABs with (c) *b* = 2 × 10^−4^ at 0.97 THz and (d) *b* = 1.5 × 10^−4^ at 0.93 THz near the focal plane. (e) Numerically simulated, Full‐wave EM simulated, and experimental normalized electric field intensity distributions *E*_*LCP*_ of EAVB with *m* = 3 at 0.93 THz.

Figure 5b shows the THz near‐field scanning setup, in which the THz signal is generated and detected by the system. The sample is mounted on a translation stage, and the transmitted field is measured with a receiving probe. The time‐domain signals are recorded at each scanning position and then converted into frequency‐domain electric fields using a fast Fourier transform. Detailed specifications of the measurement system are provided in Supporting Information S1: Section S6 of the Supplementary Materials.

Under the LP wave incidence, two RAB metasurfaces are designed by simultaneous amplitude and phase modulation, as described in Supporting Information S1: Section S8 of the Supplementary Materials. The measured fields show autofocusing behavior near *z*_1_ = 3 mm and *z*_2_ = 5 mm in Figure 5c,d, respectively. The corresponding measurement frequencies are 0.97 THz and 0.93 THz, and the experimental results agree well with the simulations.

For the CP EAVB, the metasurface designed for *m* = 3 is characterized experimentally. The transmitted fields are measured under *x*‐ and *y*‐polarized incidence separately and then combined to reconstruct the CP field distribution. The data‐processing procedure for obtaining the frequency‐domain electric field distributions from the measured time‐domain signals is provided in Supporting Information S1: Section S9 of the Supplementary Materials. As shown in Figure 5e, the measured field at 0.93 THz near *z* = 3 mm exhibits the expected left‐tilted focal topology, confirming the effective generation of EAVB by the designed metasurface. Because the required safety clearance prevents the probe from reaching the focal plane predicted in Section 3.2, the comparison focuses on the overall field morphology and the tilt of the two dominant lobes.

The measured field distributions may be affected by sample alignment, probe positioning, system noise, the finite aperture, scan step of the probe, and fabrication tolerances. These factors may contribute to deviations between the experimental results and the numerical simulations. Accordingly, the experimental results are used primarily to assess the beam morphology and spatial characteristics.

A comparison with previous studies is summarized in Table 1. Compared with related works that mainly generate basic one‐dimensional (1‐D) Airy beams or operate at microwave frequencies, the proposed composite bilayer metasurface operates in the THz regime and realizes several types of structured beams, including RAB, PRAB, and EAVB. In addition, the use of only two patterned metallic layers enables transmission/reflection dual‐channel operation together with LP/CP dual‐polarization control, improving functional integration while keeping the structural complexity manageable. This comparison highlights the capability of the present work to extend both the beam‐profile diversity and modulation dimensionality of THz Airy beam devices.

**TABLE 1:** Performance comparison with other related works.

Reference	Metal layers	Channel	Polarization	Frequency	Performance	Experiment
[41]	One	Trans.	CP	0.6 THz	1‐D Airy beams	No
[42]	One	Ref.	LP	0.9–1.2 THz	1‐D Airy beams	No
[43]	Four	Trans. and Ref.	LP and CP	17.1 GHz (CP, ref.) and 16.9 GHz (CP, ref.), 11.2 GHz (LP, trans.)	Dual‐vortex beam, Bessel beam, and polarization beam splitting	Yes
[44]	Three	Trans. and Ref.	CP	9 GHz (ref.), 13 GHz (trans.)	Lateral and axial bifocal metalenses, and multibeam	Yes
[38]	Four	Trans. and Ref.	CP	7 and 15.7 GHz (trans.), 10.2 GHz (ref.)	Dual‐vortex beams, dual focusing	Yes
[39]	Six	Trans. and Ref.	CP	8.7 GHz (trans.) and 15.8 GHz (ref.)	Hologram	Yes
[48]	Three	Ref.	LP and CP	12 and 22 GHz	Hologram	Yes
This work	Two	Trans. and Ref.	LP and CP	0.538 THz (LP, ref.), 0.553 THz (CP, ref.) and 0.99 THz (LP and CP, trans.)	RAB, PRAB, and EAVB	Yes

Abbreviations: CP, circular polarization; LP, linear polarization, Ref., reflection; Trans., transmission.

## 4 Conclusion

In summary, we propose a composite bilayer metasurface for multidimensional THz Airy beam manipulation and numerically demonstrate its functionalities in both transmission and reflection, with experimental validation of LP RAB and CP EAVB generation in the transmission channel. The metasurface combines LP amplitude‐phase modulation in the transmission channel with CP PB‐phase modulation, enabling multiple Airy beam functions to be integrated within a single compact aperture. Numerical results demonstrate the self‐healing propagation behavior of RABs and PRAB. Experimental measurements of the representative LP RAB and CP elliptical Airy vortex beam prototypes agree well with full‐wave EM simulations, verifying the feasibility of diverse Airy beam generation. In the present work, the experiments are limited to the transmission channel. Establishing this measurement capability and quantitatively validating the reflection channel performance will be an important direction for future work. This work establishes a compact and versatile platform for multi‐dimensional THz Airy‐beam manipulation for free‐space optical and 6G wireless applications.

## Author Contributions

**Yunting Bai:** conceptualization, data curation, formal analysis, investigation, methodology, software, writing – original draft. **Shaohe Li:** data curation, investigation, resources, software, validation, visualization, writing – review and editing. **Yan Wang:** conceptualization, funding acquisition, resources, supervision, project administration, writing – review and editing. **Qike Shi:** validation, visualization. **Fan Nie:** validation. **Xin Jue:** data curation, formal analysis, validation, visualization. **Yujie Liu:** methodology, resources. **Hailin Wang:** methodology. **Runfeng Su:** funding acquisition. **Jian Chen:** conceptualization, project administration.

## Funding

This work was supported by the Natural Science Foundation of Hebei Province (Grant F2026502024), Central Guidance Fund Project for Local Scientific and Technological Development of Hebei Province (Grant 264Z2101G), S&T Program of Hebei (Grant 26364503D) and National Natural Science Foundation of China (Grant 62475113).

## Supporting information


Supporting Information S1

## Data Availability

Data will be made available on request.

## References

[1] I. F. Akyildiz , C. Han , Z. Hu , S. Nie , and J. M. Jornet , “Terahertz Band Communication: An Old Problem Revisited and Research Directions for the Next Decade,” IEEE Transactions on Communications 70, no. 6 (2022): 4250–4285, 10.1109/TCOMM.2022.3171800.

[2] D. Lee , Y. Yagi , K. Suzuoki , and R. Kudo , “Experimental Demonstration of Wireless Transmission Using Airy Beams in Sub‐THz Band,” IEEE Open Journal of the Communications Society 6 (2025): 1091–1102, 10.1109/OJCOMS.2025.3534788.

[3] Z. Chen , X. Y. Ma , B. Zhang , Y. X. Zhang , Z. Q. Niu , and N. Y. Kuang , “A Survey on Terahertz Communications,” China Communications 16 (2019): 1–35, https://ieeexplore.ieee.org/document/8663550.

[4] M. S. Li , R. T. Ako , S. Sriram , C. Fumeaux , and W. Withayachumnanond , “Terahertz Planar Cavity Antenna Based on Effective Medium for Wireless Communications,” IEEE Transactions on Terahertz Science and Technology 14, no. 2 (2024): 248–257, 10.1109/TTHZ.2023.3338463.

[5] B. Ouardi , O. Koutsos , O. de Sagazan , M. Robin , R. Sauleau , and A. Clemente , “Sub‐THz Multifunctional Metasurfaces for Independent Transmission or Reflection Phase Manipulation,” Advanced Optical Materials 14, no. 4 (2026): e01768, 10.1002/adom.202501768.

[6] Y. H. Tan , K. Qu , K. Chen , et al., “Free‐Standing Single‐Layer Metasurface for Efficient and Broadband Tailoring of Terahertz Wavefront,” Advanced Optical Materials 10, no. 16 (2022): 2200565, 10.1002/adom.202200565.

[7] A. Singh , V. Petrov , P. Sen , and J. M. Jornet , “Near‐Field Terahertz Communications for 6G and Beyond: From Concepts to Realizations,” IEEE Signal Processing Magazine 42, no. 1 (2025): 106–125, 10.1109/MSP.2024.3496395.

[8] M. X. Li , J. M. Jornet , D. M. Mittleman , and C. Han , “Beam Manipulation for Terahertz Communications,” Communications Engineering 5, no. 1 (2026): 83, 10.1038/s44172-026-00676-7. PMC13139571 42082755

[9] H. A. Teng , Y. X. Qian , Y. P. Lan , and Y. M. Cai , “Abruptly Autofocusing Circular Swallowtail Beams,” Optics Letters 46, no. 2 (2021): 270–273, 10.1364/OL.415709. 33449005

[10] D. Amaya , Ó. Martínez‐Matos , and P. Vaveliuk , “Abruptly Autofocusing Beams From Phase Perturbations Having Forced Symmetry,” Optics Letters 44, no. 15 (2019): 3733–3736, 10.1364/OL.44.003733. 31368955

[11] L. Chen , B. Gao , X. Li , et al., “Optimizing Airy Needle‐Like Beams for Long‐Range Axial Manipulation and Super‐Resolution Imaging,” American Chemical Society Photonics 11, no. 9 (2024): 3610–3620, 10.1021/acsphotonics.4c00615.

[12] M. Khemiri , M. Erdem , A. Saeed , O. Gurbuz , and M. Alouini , “Robust Beam Control for Terahertz Drone Networks,” IEEE Transactions on Aerospace and Electronic Systems 61, no. 5 (2025): 13474–13487, 10.1109/TAES.2025.3581533.

[13] H. Qin , H. X. Jiang , L. L. Xu , et al., “Generating Multi‐Focus Circular Beams via All‐Dielectric Metasurfaces,” Physica Scripta 100, no. 8 (2025): 085525, 10.1088/1402-4896/adf473.

[14] H. D. L. Pires , J. Woudenberg , and M. P. van Exter , “Measurements of Spatial Coherence of Partially Coherent Light With and Without Orbital Angular Momentum,” Journal of the Optical Society of America A 27, no. 12 (2010): 2630–2637, 10.1364/JOSAA.27.002630. 21119748

[15] T. Meinert and A. Rohrbach , “Light‐Sheet Microscopy With Length Adaptive Bessel Beams,” Biomedical Optics Express 10, no. 2 (2019): 670–681, 10.1364/BOE.10.000670. PMC6377868 30800507

[16] R. A. B. Suarez , A. A. R. Neves , and M. R. R. Gesualdi , “Optical Trapping With Non‐Diffracting Airy Beams Array Using a Holographic Optical Tweezers,” Optics & Laser Technology 135 (2021): 106678, 10.1016/j.optlastec.2020.106678.

[17] S. Jia , J. C. Vaughan , and X. Zhuang , “Isotropic Three‐Dimensional Super Resolution Imaging With a Self‐Bending Point Spread Function,” Nature Photonics 8, no. 4 (2014): 302–306, 10.1038/nphoton.2014.13. PMC4224117 25383090

[18] N. Mphuthi , L. Gailele , I. Litvin , A. Dudley , R. Botha , and A. Forbes , “Free‐Space Optical Communication Link With Shape‐Invariant Orbital Angular Momentum Bessel Beams,” Applied Optics 58, no. 16 (2019): 4258–4264, 10.1364/AO.58.004258. 31251228

[19] N. Maletic , L. Lopacinski , M. Goodarzi , J. Gutierrez , and E. Grass , “A Study of LOS MIMO for Short‐Range sub‐THz Wireless Links,” in Proceedings of the 25th ITG Symposium on Mobile Communications: Technology & Applications, (2021), 1–6. https://ieeexplore.ieee.org/abstract/document/9657174.

[20] I. V. Reddy , D. Bodet , A. Singh , V. Petrov , C. Liberale , and J. M. Jornet , “Ultrabroadband Terahertz‐Band Communications With Self‐Healing Bessel Beams,” Communications Engineering 2, no. 1 (2023): 70, 10.1038/s44172-023-00118-8.

[21] M. V. Berry and N. L. Balazs , “Nonspreading Wave Packets,” American Journal of Physics 47, no. 3 (1979): 264–267, 10.1119/1.11855.

[22] G. A. Siviloglou , J. Broky , A. Dogariu , and D. N. Christodoulides , “Observation of Accelerating Airy Beams,” Physical Review Letters 99, no. 21 (2007): 213901, 10.1103/PhysRevLett.99.213901. 18233219

[23] J. Broky , G. A. Siviloglou , A. Dogariu , and D. N. Christodoulides , “Self‐Healing Properties of Optical Airy Beams,” Optics Express 16, no. 17 (2008): 12880–12891, 10.1364/OE.16.012880. 18711527

[24] A. Minovich , A. E. Klein , N. Janunts , T. Pertsch , D. N. Neshev , and Y. S. Kivshar , “Generation and Near‐Field Imaging of Airy Surface Plasmons,” Physical Review Letters 107, no. 11 (2011): 116802, 10.1103/PhysRevLett.107.116802. 22026691

[25] H. Guerboukha , B. Zhao , Z. Fang , E. Knightly , and D. M. Mittleman , “Curving THz Wireless Data Links Around Obstacles,” Communications Engineering 3, no. 1 (2024): 58, 10.1038/s44172-024-00206-3.

[26] W. Q. Zhao , S. Abadal , G. C. Song , J. M. Jiang , and C. Han , “Terahertz Wireless Data Center: Gaussian Beam or Airy Beam?,” IEEE Transactions on Wireless Communications 25 (2025): 7922–7938, 10.1109/TWC.2025.3634516.

[27] T. Latychevskaia , D. Schachtler , and H. W. Fink , “Creating Airy Beams Employing a Transmissive Spatial Light Modulator,” Applied Optics 55, no. 22 (2016): 6095–6101, 10.1364/AO.55.006095. 27505393

[28] R. A. B. Suarez , T. A. Vieira , I. S. V. Yepes , and M. R. R. Gesualdi , “Photorefractive and Computational Holography in the Experimental Generation of Airy Beams,” Optics Communications 366 (2016): 291–300, 10.1016/j.optcom.2016.01.045.

[29] X. F. Liu , Y. Zhang , Y. R. Wang , G. W. Ma , A. Xie , and Z. M. Xiong , “Electromagnetic Wave Absorption Performance of Cement‐Based Materials With Bioinspired Moth‐Eye Metasurfaces,” Materials Letters 403 (2025): 139417, 10.1016/j.matlet.2025.139417.

[30] Z. Wang , T. Lang , and Y. Qiu , “Wideband Airy Beam Generation Using Reflective Metasurfaces With Both Phase and Amplitude Modulation,” Photonics 10, no. 4 (2023): 426, 10.3390/photonics10040426.

[31] S. Zhang , P. C. Huo , Y. L. Wang , M. Li , C. Zhang , and T. Xu , “Generation of Achromatic Auto‐focusing Airy Beam for Visible Light by an All‐Dielectric Metasurface,” Journal of Applied Physics 131, no. 4 (2022): 043104, 10.1063/5.0077930.

[32] S. W. Yu , J. H. Ma , Z. C. Li , et al., “Optical‐Field Information Manipulation Based on Nonlocal Metasurfaces: From Passive to Active Systems,” Light Manipulation and Applications 1 (2026): 202612, 10.70401/lma.2026.0015.

[33] Z. C. Li , W. W. Liu , Y. B. Zhang , H. Cheng , S. Zhang , and S. Q. Chen , “Optical Polarization Manipulations With Anisotropic Nanostructures,” PhotoniX 5, no. 1 (2024): 30, 10.1186/s43074-024-00143-6.

[34] C. L. Zheng , J. Li , G. C. Wang , et al., “Fine Manipulation of Terahertz Waves via All‐Silicon Metasurfaces With an Independent Amplitude and Phase,” Nanoscale 13, no. 11 (2021): 5809–5816, 10.1039/d1nr00376c. 33710236

[35] C. L. Zheng , J. Li , G. C. Wang , et al., “All‐Dielectric Metasurfaces Capable of Dual‐Channel Complex Amplitude Modulation,” Nanophotonics 10, no. 11 (2021): 2959–2968, 10.1515/nanoph-2021-0349.

[36] P. Zhang , B. Fang , T. Q. Zhao , et al., “Amplitude and Phase Independent Modulation Based on Transmission‐Type Metasurfaces,” Results in Physics 46 (2023): 106328, 10.1016/j.rinp.2023.106328.

[37] T. Liu , C. H. Dai , W. K. Feng , et al., “Arbitrary Orthogonal Polarization Decomposition and Routing With Complex Amplitude Modulation via Wheel‐of‐Fortune‐Inspired Metasurfaces,” Advanced Science 13, no. 30 (2026): e00046, 10.1002/advs.202600046. PMC13248823 41841593

[38] H. X. Xu , J. Xu , Y. Z. Wang , C. H. Wang , F. Zhang , and G. W. Hu , “Multichannel Metasurfaces With Frequency‐Direction Multiplexed Amplitude and Phase Modulations,” Advanced Optical Materials 11, no. 22 (2023): 2301299, 10.1002/adom.202301299.

[39] C. H. Wang , H. X. Xu , T. Liu , and F. Zhang , “Hybrid‐Phase Assisted Amplitude and Phase Control Using Full‐Space Metasurface,” Advanced Optical Materials 12, no. 10 (2024): 2302153, 10.1002/adom.202302153.

[40] H. X. Xu , G. W. Hu , L. Han , et al., “Chirality‐Assisted High‐Efficiency Metasurfaces With Independent Control of Phase, Amplitude, and Polarization,” Advanced Optical Materials 7, no. 4 (2019): 1801479, 10.1002/adom.201801479.

[41] K. L. Xi , B. Fang , L. Ding , L. Li , S. L. Zhuang , and Q. Q. Cheng , “Terahertz Airy Beam Generated by Pancharatnam‐Berry Phases in Guided Wave‐Driven Metasurfaces,” Optics Express 30, no. 10 (2022): 16699–16711, 10.1364/oe.456699. 36221507

[42] Z. S. Zhou , R. X. Zhang , Z. J. Chen , R. X. Nie , Z. M. Pan , and Z. Y. Song , “Terahertz Generation of Airy Beam With Polarization Conversion in a Reflective Vanadium Dioxide Metasurface,” Physica Scripta 99, no. 5 (2024): 055555, 10.1088/1402-4896/ad3ee8.

[43] H. L. Luo , H. H. Gao , Y. Z. Wang , et al., “Multichannel Full‐Space Coding Metasurface With Linearly‐Circularly‐Polarized Wavefront Manipulation,” Nanophotonics 13, no. 22 (2024): 4169–4179, 10.1515/nanoph-2024-0331. PMC11501062 39635443

[44] X. M. Li , Y. Zhao , R. P. Lu , et al., “Full‐Space Trifunctional Metasurface With Independent Control of Amplitude and Phase for Circularly Polarized Waves,” Nanophotonics 13, no. 24 (2024): 4471–4481, 10.1515/nanoph-2024-0441. PMC11636479 39679187

[45] H. Y. Zhang , N. Shlezinger , F. Guidi , D. Dardari , M. F. Imani , and Y. C. Eldar , “Beam Focusing for Near‐Field Multiuser MIMO Communications,” IEEE Transactions on Wireless Communications 21, no. 9 (2022): 7476–7490, 10.1109/TWC.2022.3158894.

[46] H. Y. Zhang , N. Shlezinger , F. Guidi , D. Dardari , and Y. C. Eldar , “6G Wireless Communications: From Far‐Field Beam Steering to Near‐Field Beam Focusing,” IEEE Communications Magazine 61, no. 4 (2023): 72–77, 10.1109/MCOM.001.2200259.

[47] Y. Guo , M. Ansari , R. W. Ziolkowski , and N. J. G. Fonseca , “Quasi‐Optical Multi‐Beam Antenna Technologies for B5G and 6G mmWave and THz Networks: A Review,” IEEE Open Journal of Antennas and Propagation 2 (2021): 807–830, 10.1109/OJAP.2021.3093622.

[48] Y. Gou , H. F. Ma , L. W. Wu , S. Sun , Z. X. Wang , and T. J. Cui , “Non‐Interleaved Polarization‐Frequency Multiplexing Metasurface for Multichannel Holography,” Advanced Optical Materials 10, no. 22 (2022): 2201142, 10.1002/adom.202201142.

