# Supplementary material for: Full‐Space Terahertz Airy Beam Shaping via Composite Bilayer Metasurface With Switchable Amplitude–Phase Encoding Channels
Source: Nanophotonics. 2026 Oct 31;15(21):e70351. doi: 10.1002/nap2.70351 (PMC13722953; doi:10.1002/nap2.70351)
Supplement: Supplementary file 1 [file NAP2-15-e70351-s001.docx]

Supplementary Materials

**Full-Space Terahertz Airy Beam Shaping via Composite Bilayer Metasurface with Switchable Amplitude-Phase Encoding Channels**

*Yunting Bai, Shaohe Li, Yan Wang*, Qike Shi, Fan Nie, Xin Jue, Yujie Liu, Hailin Wang, Runfeng Su, and Jian Chen*

Yunting Bai, Yan Wang, Qike Shi, Fan Nie, Xin Jue
Yanzhao Electric Power Laboratory of North China Electric Power University, North China Electric Power University, Baoding, Hebei, 071003, China
Hebei Engineering Research Center of Intelligent Technology for Power Internet of Things, North China Electric Power University, Baoding, Hebei, 071003, China
E-mail: [wyyd2016@ncepu.edu.cn](mailto:wyyd2016@ncepu.edu.cn)

Shaohe Li, Runfeng Su, Jian Chen
Research Institute of Superconductor Electronics, School of Electronic Science and Engineering & National Key Laboratory of Microwave Photonics (Nanjing University), Nanjing University, Nanjing, Jiangsu, 210023, China

Yujie Liu
National Key Laboratory of Microwave Photonics, College of Electronic and Information Engineering, Nanjing University of Aeronautics and Astronautics, Nanjing 211106, China

Hailin Wang
State Key Laboratory of Millimeter Waves, Southeast University, Nanjing 210096, China

**Contents**

S1. Theoretical analysis of linear- and circular-modulation mechanisms of the meta-atom

S2. The isolation between the transmission and reflection channels

S3. Effects of the topological charge and phase-modulation parameter on the focal plane distributions of multi-point ring Airy beams

S4. Direction and magnitude of focal tilt determined by topological charge *m*

S5. Sample fabrication process

S6. THz time-domain spectroscopy system and THz near-field scanning system

S7. Schematic of two orthogonal sample orientations for *α*_2_ = -45° and 45°

S8. The detailed design process for amplitude- and phase- modulated ring Airy beam metasurface

S9. Data processing procedure for CP reconstruction

**S1. Theoretical analysis of linear- and circular-polarization modulation mechanisms of the meta-atom**

The polarization response of the meta-atom can first be described in the linear polarization (LP) basis by the Jones matrix.

$J^{lin}=\left(\begin{matrix} A_{xx} & A_{xy} \\ A_{yx} & A_{yy} \end{matrix}\right)$ (S1)

where *A_ij_* denotes the complex transmission or reflection coefficient, depending on the operating channel, and *i*, *j* ϵ {*x*, *y*}. For an *x*-polarized incident wave, the output field can be expressed as

$\left[\begin{matrix} E_{x}^{\text{out}} \\ E_{y}^{\text{out}} \end{matrix}\right]=\left[\begin{matrix} A_{xx} \\ A_{yx} \end{matrix}\right]E_{x}^{in}$ (S2)

Accordingly, the LP modulation behavior is mainly governed by the cross-polarized coefficient *A_yx_*, which can be written as

(S3)

For a rotated anisotropic meta-atom, the Jones matrix satisfies

$J^{lin}\left(\alpha\right)=R\left(-\alpha\right)J^{lin}\left(0\right)R\left(\alpha\right)$ (S4)

Where *R*(*α*) is the in-plane rotation matrix and *α* is the rotation angle. If the principal-axis responses are denoted by *A_u_* and *A_v_*, the cross-polarized coefficient under *x*-polarized incidence can be expressed as

$A_{yx}=\frac{1}{2}\left(A_{u}-A_{v}\right)\sin2\alpha$ (S5)

This relation indicates that the amplitude of the cross-polarized component follows a sin2*α* type dependence on the rotation angle. When the principal-axis components exhibit nearly equal amplitudes but an approximately 180° phase difference, the meta-atom can simultaneously provide continuous amplitude modulation and binary phase switching in the linear cross-polarized channel. In the present design, this LP modulation is implemented by rotating the C-shaped aperture through the angle *α*_1_ in the reflection channel and by rotating the Φ-shaped structure through the angle *α*_2_ in the transmission channel.

For circular polarization (CP) incidence, the modulation behavior is PB phase. In the ring polarization basis, the output field of a rotated anisotropic meta-atom can be decomposed into a co-polarized component and a cross-polarized component. For an incident CP wave $E_{\sigma}^{in}$ the output field can be written as

$E^{out}=\frac{A_{u}+A_{v}}{2}E_{\sigma}^{in}+\frac{A_{u}-A_{v}}{2}e^{\pm i2\alpha}E_{-\sigma}^{out}$ (S6)

Where *σ* denotes the handedness of the incident wave and $E_{-\sigma}^{out}$ represents the cross-polarized component orthogonal to the incident polarization state. When the conditions $\left|A_{u}\right|\approx\left|A_{v}\right|$ and $\left|\arg\left(A_{u}\right)-\arg\left(A_{v}\right)\right|\approx180^{\circ}$ are satisfied, the co-polarized term is strongly suppressed. Under this condition, Equation (S6) can be simplified as

$E^{out}=\frac{A_{u}-A_{v}}{2}e^{\pm i2\alpha}E_{-\sigma}^{out}$ (S7)

Equation (S7) directly shows that the phase of the cross-polarized output follows the PB phase relation *Φ*_PB =_ ±2*α*. Therefore, continuous phase control can be achieved simply by rotating the meta-atom.

In the proposed design, the above LP and CP modulation mechanisms are implemented in different operating channels through the rotation of the substructures constituting the composite meta-atom. The corresponding channel-dependent responses are further verified by the simulated amplitude and phase characteristics presented in the main text.

The polarization-conversion ratio of the meta-atom is defined as:

$PCR=\frac{\left|S_{cross}\right|^{2}}{\left|S_{cross}\right|^{2}+\left|S_{co}\right|^{2}}\times100\%$ (S8)

where *Scross* and *Sco* represent the cross-polarized and co-polarized transmission or reflection coefficients, respectively, for the corresponding LP or CP incident wave.

The total transmitted or reflected power in the half-space is calculated as:

$P_{total}=(\left|S_{cross}\right|^{2}+\left|S_{co}\right|^{2})\times100\%$ (S9)

The bandwidth is evaluated using the criteria |*Scros*s|² ≥ 50% and PCR ≥ 80%*.*

**S2. The isolation between the transmission and reflection channels**

As seen in **Figure S1**, the strong surface currents are consistently concentrated in the substructures responsible for the corresponding operating channel. At *f*₁ = 0.99 THz, the dominant currents are mainly distributed on the Φ-shaped metallic structures on the top and bottom layers, whereas the currents on the C-shaped aperture are comparatively weak. In contrast, at *f*_2_ = 0.538 THz and *f*_3_ = 0.553 THz, the strong currents are mainly localized around the top-layer C-shaped aperture, while the other regions exhibit much weaker current distributions and thus contribute less to the overall resonant response.

**Figure S1.** Representative surface current distributions of the proposed composite meta-atom under four operating frequencies. (a) Transmission channel at *f*₁ = 0.99 THz under right circularly polarized (RCP) incidence. (b) Reflection channel at *f*_3_ = 0.553 THz under RCP incidence. (c) Transmission channel at *f*₁ = 0.99 THz under *x*-polarized incidence. (d) Reflection channel at *f*_2_ = 0.538 THz under *x*-polarized incidence.

These results indicate that the transmission and reflection channels are dominated by different substructures at different operating frequencies. The transmission response at *f*₁ is mainly associated with the Φ-shaped structures, whereas the reflection responses at *f*_2_ and *f*_3_ are mainly associated with the C-shaped aperture. Such a current redistribution pattern supports the interpretation that the two channels are sustained by different resonant pathways.

The isolation between the transmission and reflection channels is further verified in **Figure S2**. The rotation angles are set as *α*₂ = 0° and 90°, and *α*₁ = -45°, 0°, and 45°. As shown in Figure S2(a), the transmission amplitude and phase at *f*₁ is sensitive to *α*₂ but only weakly affected by *α*₁. In contrast, Figure S2(b) shows that the cross-polarization reflected wave at *f*₂ is strongly dependent on the rotation angle *α*₁ of the C-shaped aperture and is insensitive to *α*₂. These results indicate that PB phase modulation in the transmission channel is governed by rotating the Φ-shaped structure under CP incidence, while amplitude-phase modulation in the reflection channel is controlled by rotating the C-shaped aperture under LP incidence. The weak mutual influence between the two channels indicates low crosstalk and supports their independent operation.

**Figure S2**. Verification of the isolation between the transmission and reflection channels:(a) transmission amplitude and phase at *f*₁ under different *α*₂ and *α*₁; (b) cross-polarized reflection amplitude and phase at *f*₂ under different *α*₁ and *α*₂.

**S3.** **Effects of the topological charge and phase-modulation parameter on the focal plane distributions of multi-point ring Airy beams**

To further clarify the formation mechanism of the multi-point ring Airy beams and the rationale for parameter selection, **Figures S3-S6** present the normalized electric-field intensity distributions at the focal plane under different topological charges and phase-modulation parameters.

**Figure S3.** Normalized electric-field intensity distributions at the focal plane of the three-point ring Airy beam with different topological charges: (a) *m* = 1, (b) *m* = 2, and (c) *m* = 3.

**Figure S4.** Normalized electric-field intensity distributions at the focal plane of the four-point ring Airy beam with different topological charges: (a) *m* = 3, (b) *m* = 4, and (c) *m* = 5.

As shown in Figures S3 and S4, keeping the other parameters unchanged and increasing the topological charge enlarges the central hollow region and improves the separability of the focal points for both the three-point and four-point ring Airy beams; however, excessively large values of *m* also tend to deteriorate the energy uniformity among the points. For the four-point case, *m* = 5 provides a suitable compromise under the present aperture condition, because it yields clear four-point splitting while maintaining relatively concentrated and uniform energy distribution, together with comparatively weak intermediate sidelobes.

**Figure S5.** Normalized electric-field intensity distributions at the focal plane of the three-point ring Airy beam with different phase-modulation parameters: (a) *δ* = -10π, (b) -15π, and (c) -20π.

**Figure S6.** Normalized electric-field intensity distributions at the focal plane of the four-point ring Airy beam with different phase-modulation parameters: (a) *δ* = -10π, (b) -20π, and (c) -30π.

Figure S5 and S6 further show that the phase-modulation parameter strongly affects the splitting behavior. For the three-point beam, the points remain partially overlapped at *δ* = -10π and -15π, whereas a clearly resolved three-point pattern is obtained at -20π, indicating that stronger azimuthal phase modulation is required to produce sufficient lateral separation. For the four-point beam, splitting can already be observed at -10π because the larger topological charge has already expanded the central hollow region; however, as the absolute value of the phase modulation parameter further increases, the sidelobes between adjacent focal points become more pronounced. Therefore, the different behaviors observed in the three-point and four-point cases are reasonable, and the parameter combination adopted in the main text represents a compromise between focal-point resolvability and sidelobe suppression. \

**Table S1. Qualitative comparison of the effects of *δ* and *m* on focal-point separation, energy uniformity, and sidelobe level.**

|  | ***δ*** | ***m*** | **Point separation** | **Energy uniformity** | **Sidelobe**  **level** |
| --- | --- | --- | --- | --- | --- |
| **three-point ring Airy beam** | **-20π** | **1** | **Clearly resolved** | **Good** | **Weak** |
|  |  | 2 | Clearly resolved | Poor | Moderate |
|  |  | 3 | Clearly resolved | Poor | Weak |
|  | -15π | 1 | Overlapped | Moderate | Weak |
|  | -10π |  | Overlapped | poor | Strong |
| **four-point ring Airy beam** | **-10π** | 3 | Clearly resolved | Moderate | Moderate |
|  |  | 4 | Partially resolved | Moderate | Moderate |
|  |  | **5** | **Clearly resolved** | **Good** | **Weak** |
|  | -20π |  | Clearly resolved | Good | Moderate |
|  | -30π |  | Clearly resolved | Good | Strong |

**Figure S7.** Comparison between the theoretical calculation and the full-wave simulation of the phase-modulated ring Airy beams at representative propagation distances. (a)-(f) Three-point Airy beam at *z* = −0.6 mm, −1.2 mm, −2.0 mm, 0.6 mm, 2.1 mm, and 2.5 mm, respectively. (g)-(l) Four-point Airy beam at *z* = −0.6 mm, −1.2 mm, −2.0 mm, 0.6 mm, 2.1 mm, and 2.5 mm, respectively.

As qualitatively summarized in **Table S1**, the final splitting performance of the multi-point ring Airy beams is not determined by a single parameter, but jointly governed by the phase-modulation parameter δ, the topological charge *m*, and the finite aperture of the metasurface. For the three-point PRAB, *δ* = -20π provides sufficient lateral separation, while *m*_3_ = 1 maintains good energy uniformity and weak sidelobes. For the four-point PRAB, the combination of *m*₄ = 5 and *δ* = −10π provides a suitable balance between focal-point resolution, energy distribution, and sidelobe suppression. Specifically, larger values of |*δ|* can increase focal-point separation but may also introduce stronger intermediate sidelobes under the finite-aperture condition. Therefore, *δ* = −10π is selected to suppress these unwanted sidelobe components. Based on the systematic parameter analysis, the combinations (*δ*, *m*_3_) = (-20π, 1) and (*δ*, *m*_4_) = (-10π, 5) are selected as compromises among focal-point resolvability, energy uniformity, and sidelobe suppression under the present finite-aperture condition.

The focusing efficiencies of the transmission and reflection PRABs are calculated from the simulated electric field distributions. The intensities of the total electric field and the target-polarization component are defined as follows:

$I_{total}=\left|E_{x}\right|^{2}+\left|E_{y}\right|^{2}+\left|E_{z}\right|^{2}$ (S10)

$I_{t\arg et}=\left|\frac{E_{x}+iE_{y}}{\sqrt{2}}\right|^{2}$ (S11)

For each focal point, the radius of the circular integration aperture is set to three times the full width at half maximum (FWHM). The focusing efficiencies are calculated as:

$\eta=\frac{\iint I_{t\arg et}dS_{foc}}{\iint I_{ref}dS_{ref}}\text{×}100\%$ (S12)

*S_foc_* is the circular integration aperture. *I_ref_* denotes the mean total-field intensity on the focus plane without the metasurface, and *S_ref_* is the area of that focus plane.

**S4. Direction and magnitude of focal tilt determined by topological charge *m***

**Figure S8** theoretically investigates the relationship between the focal tilt and the topological charge m. The normalized focal-plane intensity distributions for *m* = +2, -2, +3, -3, +4, and -4 are presented in Figure S8.

The results show that the tilt direction is determined by the sign of *m*. When *m* > 0, the focal pattern exhibits a leftward tilt from bottom to top, whereas for *m* < 0, the tilt direction is reversed. For instance, in the case of *m* = +2, the upper focal point is shifted to the left and the lower one to the right, leading to an overall leftward tilt; in contrast, the pattern for *m* = -2 shows the opposite trend. In addition to the tilt direction, the tilt angle is also affected by the magnitude of the topological charge.

As |*m*| increases from 2 to 4, the calculated electric-field tilt angles increase from 9.64° to 10.92° and 21.61°, respectively, indicating that a larger topological charge leads to a more pronounced focal tilt. These results confirm that the sign of *m* governs the tilt direction, while its magnitude determines the degree of tilt.

**Figure S8.** Theoretical validation of the focal tilt characteristics determined by the topological charge. The theoretically calculated intensity profiles on the focal plane for topological charges of (a) *m* = +2 (left) and *m* = -2 (right), (b) *m* = +3 (left) and *m* = -3 (right),(c) *m* = +4 (left) and *m* = -4 (right), respectively.

**S5. Sample fabrication process**

All samples consist of 55×55 meta-atoms. To mitigate fabrication-induced dimensional errors, the Φ-shaped structure is modified from a varying-width design to one with a uniform arm width. The fabrication of the composite bilayer metasurface sample primarily involves two main procedures: (1) the preparation of polyimide layers with varying thicknesses via spin-coating and thermal curing, and (2) the formation of metallic structures using standard photolithography, magnetron sputtering, and a lift-off process.

**S5.1 Polyimide Precursor Treatment (Bottom Supporting Layer)**

A polyimide precursor is first spin-coated onto a silicon substrate. It is then subjected to a stepwise thermal curing process: 30 min at 80 °C, followed by 1 hour each at 120 °C, 170 °C, and 220 °C, and finally 2 hours at 320 °C. This process yields a polyimide supporting layer with a thickness of approximately 5 μm. The spin-coating and thermal curing parameters are summarized in **Table S2** and **S3**.

**Table S2. Spin-coating parameters**

|  | **Spin speed/rpm** | **Time/s** | **Acceleration /(rpm/s)** |
| --- | --- | --- | --- |
| 1 | 600 | 9 | 600 |
| 2 | 3200 | 40 | 4000 |

**Table S3. Thermal curing parameters**

|  | **Temperature/℃** | **Times/min** |
| --- | --- | --- |
| 1 | 80 | 30 |
| 2 | 120 | 60 |
| 3 | 170 | 60 |
| 4 | 220 | 60 |
| 5 | 320 | 120 |

**S5.2 Fabrication of the bottom metallic structure**
 A bilayer photoresist (LOR10B + AZ1500) is spin-coated onto the polyimide supporting layer and thermally cured. The spin-coating parameters for each cycle are as follows: **Table S4.** **Spin-coating parameters**

|  | **Spin speed/rpm** | **Time/s** |
| --- | --- | --- |
| 1 | 600 | 6 |
| 2 | 4000 | 40 |

The bottom metallic structure is patterned using standard photolithography and a lift-off process. First, a bilayer photoresist (LOR10B + AZ1500) is spin-coated onto the polyimide supporting layer and thermally cured. The sample is then exposed to UV light for 7 s and developed in ZXJ-100 developer for 7 s to define the structure pattern. Subsequently, a 10-nm-thick Ti adhesion layer followed by a 200-nm-thick gold layer are deposited via magnetron sputtering. The final metallic pattern is realized via the following lift-off process:

1). The sample is immersed in acetone and ultrasonicated at 50% power for 5-7 min to remove the excess metal.

2). It is then transferred to an alcohol solution for an additional 2 min of ultrasonication.

3). The sample is rinsed with deionized (DI) water.

4). Residual photoresist is removed using ZXJ-100 developer for approximately 12 s.

5). The sample is rinsed again with DI water and dried using a nitrogen gun.

**S5.3 Fabrication of the middle polyimide layer**

Following the completion of the bottom metallic structure, the middle polyimide layer is formed by repeating the spin-coating and curing process as described in Section S5.1 five times. Each cycle adds a thickness of approximately 10 μm, yielding a total intermediate-layer thickness of approximately 50 μm. The spin-coating parameters for each cycle are as follows:

**Table S5. Spin-coating parameters**

|  | **Spin speed/rpm** | **Time/s** | **Acceleration /(rpm/s)** |
| --- | --- | --- | --- |
| 1 | 600 | 9 | 600 |
| 2 | 1500 | 40 | 4000 |

**S5.4 Fabrication of the top metallic structure**

Due to experimental equipment limitations, aluminum is used instead of gold for the fabrication of the top metallic structure. The photoresist spin-coating and photolithographic patterning processes described in Section S5.2 are repeated for the top metallic layer. Subsequently, a 0.2 μm thick Aluminum (Al) film is deposited using a RF magnetron sputtering system operated at an RF power of 150W, an Ar flow rate of 70 sccm, a chamber pressure of 5×10^-6^ Torr, and a substrate temperature of 18 °C. Finally, the patterned top Al layer is obtained through a lift-off process.

**Figure S9.** Sample fabrication process

**Figure S10.** Amplitude and phase responses of the modified meta-atom under *x*-polarized wave incidence in the (a,b) reflection and (c,d) transmission channels.

**Figure S11**. Amplitude and phase responses of the modified meta-atom under RCP wave incidence in the (a,b) reflection and (c,d) transmission channels.

Additional simulations were performed to evaluate the effects of replacing the top Au layer with Al and modifying the resonator to have a uniform arm width, as shown in Figs. S10 and S11. In the simulations, Al is modeled as a lossy metal with an electrical conductivity of σ = 3.56×10⁷ S/m. The amplitude and phase responses of the modified meta-atom under *x*-polarized and RCP wave incidence are presented in Figures S10 and S11, respectively. The results show that the redesigned resonator preserves the required amplitude and phase modulation characteristics in Figure 2, while the uniform arm width simplifies the geometry and improves its tolerance to fabrication deviations.

**S6.** **THz time-domain spectroscopy system and THz near-field scanning system**

The S-parameters of the meta-atom are characterized using a THz time-domain spectroscopy system as shown in Figure S12. The THz radiation is generated and detected by system-integrated photoconductive antennas operating over 0.1–1 THz. The complex transmission response is obtained by Fourier transforming the measured time-domain waveforms and normalizing the sample spectrum to a free-space reference spectrum. The reference spectrum is measured without the sample under identical alignment, optical-path, and measurement conditions to minimize systematic errors.

**Figure S12**. THz time-domain spectroscopy system

The generated beam distributions are measured using a THz near-field scanning system from Botehertz Company. Unlike conventional microwave near-field measurements, the transverse electric field is detected using an all-fiber-coupled photoconductive probe. The probe integrated into the measurement system operates over 0.1–2 THz, provides a spectral resolution of 2.5 GHz, and offers a spatial resolution of 20–50 μm. The probe aperture dimension is not specified by the manufacturer.

**S7. Schematic of two orthogonal sample orientations for *α*_2_ = -45° and 45°**

**Figure S14.** Two orthogonal sample orientations, corresponding to the cases of *α*_2_ = -45°and 45°

**S8. The detailed design process for amplitude- and phase- modulated ring Airy beam metasurface**

**Figure S15.** (a) The detailed design process for amplitude and phase modulated ring Airy beam metasurface. (b) Target amplitude and phase distribution required for the ring Airy beam metasurface wavefront. (c) The rotation angles of the meta-atom in the ring Airy beam metasurfaces.

As illustrated in **Figure S15(a)**, the detailed design process for amplitude and phase modulated ring Airy beam metasurface is presented as follows. First, a parameter sweep of the Φ-shaped resonant unit is performed over a rotation angle range from -45° to 45°, yielding 101 sets of unit response data. Next, based on the obtained amplitude responses, a fitting process is carried out in MATLAB using the function model *y  =  A*sind(2*α* + *b*) + *c*, establishing a mapping relationship between the rotation angle and transmission amplitude. Subsequently, according to the target amplitude and phase distribution required for the wavefront (Figure S15(b)), the corresponding theoretical rotation angles for each unit are derived by inverting the fitted model. In this design, the position of the maximum electric field amplitude of the ring Airy beams is controlled by tuning the scale parameter *b*. We select two values: *b*_1_ = 1.5×10 ^-4^ and *b*_2_ = 2×10 ^-4^, respectively. The other parameters are fixed at *a* = 0.001, *r*_0_ = 7.5×10^-4^. Finally, the theoretical angles are matched with the fitting results to determine the actual rotation angles of the units (Figure S15(c)), and the metasurface array is automatically generated and subjected to full-wave electromagnetic simulation using a joint CST Studio Suite and MATLAB approach.

**S9.** **Data processing procedure for CP reconstruction**

The experimentally measured amplitude and phase distributions of the linear-polarization transmission coefficients are first imported for the *xx*, *yy*, and *yx* channels at the target frequency. To compensate for the sample orientation during measurement, the datasets of the rotated channels are corrected by matrix rotation. The phase data are then converted from degrees to radians. Based on the measured amplitude and phase, the complex Jones matrix elements are reconstructed.

In the present processing, the missing *t_xy_* term is approximated by *t_yx_*. This approximation is supported by the simulated reciprocity-related response of the fabricated unit, where the amplitude and phase differences between *t_xy_* and *t_yx_* remain sufficiently small near the operating frequency. The influence of this approximation mainly affects weak sidelobe details and does not change the dominant tilted-lobe morphology used for experimental validation.

Since the measured matrices do not always have identical dimensions, all complex Jones-matrix elements are further resized to a common grid by separately interpolating their real and imaginary parts and then recombining them into complex matrices. Due to the limited availability of the measurement system, the original near-field scans are acquired with a scan step of 500 μm, resulting in field maps with relatively coarse spatial sampling. To improve the presentation of the measured electric field, the complex field matrices are further processed by two-dimensional cubic interpolation and resized onto a unified 100×100 grid. This treatment avoids phase artifacts caused by directly interpolating wrapped phase distributions. A weak Gaussian smoothing is applied in the complex field domain before plotting the amplitude distributions. These interpolation and smoothing procedures are used only for visualization enhancement and do not alter the dominant spatial features or physical conclusions extracted from the original measurements. After obtaining the aligned linear-basis Jones matrix, the transmission coefficients in the CP basis are calculated through the standard linear-to-ring basis transformation. In particular, the cross-handed transmission components *t_LR_* are obtained from the measured linear-polarization components. The interpolated complex fields are subsequently used to calculate the amplitude and phase distributions in the CP basis.
